# Inbreeding depression in diploid potato: genetic basis, deleterious load, and breeding mitigation

**DOI:** 10.3389/fpls.2026.1883768

**Published:** 2026-07-08

**Authors:** Yiqian Wang, Tuanrong Lin, Wei Wang, Yufeng Wang, Longqiu Fan, Wenjuan Huang, Xinlei Jiao, Zhen Wang, Haolei Wu, Xiaojie Zong, Anke Zheng, Fan Yang, Bingxue Zhang, Jie Yu, Junzhi Chen, Yuhe Yin

**Affiliations:** 1 Ulanqab Academy of Agriculture and Forestry Sciences, Inner Mongolia Potato Technology Innovation Center, Ulanqab, Inner Mongolia, China; 2 College of Horticulture and Plant Protection, Inner Mongolia Agricultural University, Hohhot, Inner Mongolia, China; 3 Forestry Workstation, Forestry and Grassland Administration of Ulanqab City, Ulanqab, Inner Mongolia, China

**Keywords:** deleterious load, diploid potato, hybrid breeding, inbreeding depression, structural variation

## Abstract

The development of diploid hybrid potato derived from traditional tetraploid clonal cultivars is a key breakthrough for modern potato breeding. However, severe inbreeding depression severely restricts the breeding and application of homozygous inbred lines. This narrative review systematically elaborates the phenotypic characteristics of inbreeding depression in diploid potato, and explores the accumulation mechanism of deleterious load driven by long-term clonal propagation and polyploid genetic buffering. Continuous selfing elevates genome-wide homozygosity, which exposes recessive deleterious variants and further causes comprehensive declines in plant vigor, reproductive capacity, tuber yield, tuber quality and stress tolerance. We then summarize multiple quantitative evaluation systems for inbreeding depression and genetic load, and sort out a series of targeted breeding interventions, including genomic prediction, marker-assisted selection, CRISPR-Cas9 genome editing and hybrid complementation. Each technical strategy is discussed with explicit discussion of its practical limitations. Finally, we discuss the research prospects of genome design breeding for purging deleterious variants, and distinguish laboratory research progress from large-scale industrial application. This review establishes an explicit logical framework linking deleterious load, genomic homozygosity, phenotypic deterioration and breeding strategies, and provides theoretical references for the innovation of diploid potato inbred line breeding.

## 1 Introduction

Potato (*Solanum tuberosum* L.) is one of the most important staple crops worldwide, with annual production exceeding 370 million tons (Food and Agriculture Organization of the United Nations, 2024), and it plays a pivotal role in global food security and agricultural sustainability (Devaux et al., 2021; Song et al., 2024). Conventional cultivated potato is an autotetraploid with extreme heterozygosity and tetrasomic inheritance, which complicates genetic dissection, prolongs breeding cycles to 8–12 years, and reduces the efficiency of trait pyramiding. To address these challenges, diploid hybrid breeding has been proposed, aiming to establish an inbred-line-based system similar to rice and maize, shortening the breeding cycle to 2–3 years and enhancing selection efficiency (Lindhout et al., 2011; Jansky et al., 2016).

Two major obstacles limit diploid potato breeding: self-incompatibility (SI) and inbreeding depression (ID). In recent years, identification of the *Sli* gene and CRISPR-Cas9 editing of S-RNase have largely overcome SI, enabling stable production of fertile seeds via selfing (Ye et al., 2018). However, severe ID remains the most critical bottleneck for developing highly homozygous inbred lines and elite hybrids (Zhang et al., 2019, 2021).

ID is a common genetic phenomenon in outcrossing species, and it is particularly severe in clonally propagated crops (Ramu et al., 2017). The adverse effects of selfing on plant fitness were first observed by Darwin in the 19th century, and this finding has been continuously interpreted and supplemented by modern genetic studies (Charlesworth and Willis, 2009). Long-term outcrossing, clonal reproduction and ancient polyploidization have led to extensive accumulation of recessive deleterious variants in potato. These variants are masked in heterozygous states, while selfing-induced homozygosity will fully expose their harmful effects (Zhang et al., 2019). Polyploidy further promotes the accumulation of genetic load, and traditional tetraploid potato has become a major reservoir of recessive deleterious variants. When breeders develop diploid inbred lines from traditional tetraploid potato germplasm, the recessive deleterious load masked by polyploid genetic buffering is fully exposed, thereby exacerbating inbreeding depression (Lian et al., 2019). Similar patterns of deleterious load accumulation have also been documented in other clonal crops such as cassava (Ramu et al., 2017); however, the polyploid buffering mechanism unique to tetraploid potato leads to distinct load accumulation characteristics, so these evolutionary rules can only be used as indirect cross-reference rather than direct inference for potato. With the advances in pan-genomics and multi-omics technologies, research on potato ID has shifted from simple phenotypic observation to genome-wide precise dissection (Zhang et al., 2021; Wu et al., 2023).

**Figure 1:**
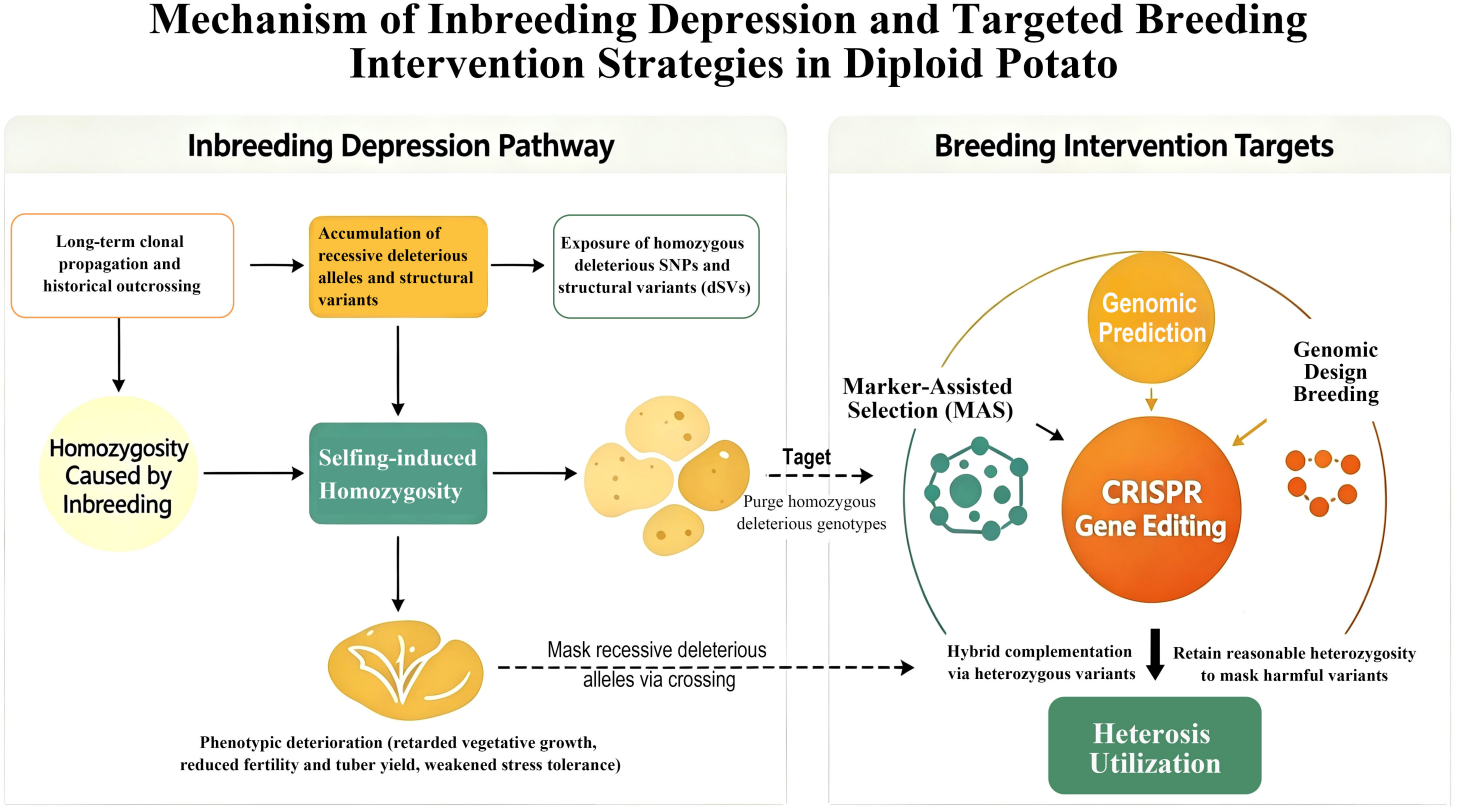
Schematic diagram illustrating the formation pathway of inbreeding depression and corresponding targeted breeding improvement strategies in diploid potato. Left panel: Long-term clonal propagation accumulates recessive deleterious SNPs and structural variants (dSVs), while continuous selfing elevates genome-wide homozygosity. The homozygous exposure of harmful variants ultimately leads to multi-dimensional phenotypic deterioration including stunted growth, low fertility, yield loss and weakened stress resistance. Right panel: Integrated breeding intervention toolkit to mitigate inbreeding depression. Marker-assisted selection (MAS), genomic prediction and CRISPR gene editing are applied to purge homozygous deleterious genotypes; heterosis utilization via inter-line crossing maintains moderate heterozygosity to mask recessive harmful alleles, realizing hybrid complementation.

Numerous studies have focused on the phenotypes, genetic mechanisms, detection methods and mitigation strategies of potato inbreeding depression. In this review, we organize relevant findings in a progressive order: phenotypic performance, genetic and molecular mechanisms, evaluation approaches, and practical breeding solutions. We construct a complete research chain from the origin of ID to targeted improvement measures, so as to provide systematic theoretical guidance for efficient diploid potato breeding. The overall formation mechanism and corresponding breeding intervention strategies for inbreeding depression are illustrated in **Figure 1**.

### 1.1 Literature search and selection strategy

This manuscript is defined as a narrative review. Literature retrieval was implemented across three mainstream academic databases: Web of Science Core Collection, Google Scholar and CNKI. Combined retrieval keywords were: (diploid potato OR Solanum tuberosum diploid) AND (inbreeding depression OR genetic load OR deleterious variant OR structural variation) AND (hybrid breeding OR genome editing). The time scope prioritized peer-reviewed original articles, formal reviews and preprints published from 2016 to May 2026; classic foundational papers (De Jong and Rowe, 1971; Potato Genome Sequencing Consortium, 2011) were supplemented to complete theoretical context. Exclusion criteria included conference abstracts lacking complete experimental datasets and research solely focused on tetraploid potato agronomic traits with no discussion of inbreeding depression. Studies focusing on non-potato clonal/model crops (cassava, grape, rice) were merely retained for comparative background discussion; all cross-species evolutionary conclusions are strictly separated from potato experimental results in the text, and we never extrapolate crop-specific genetic mechanisms of non-potato species to potato to avoid confusing interspecific differences.

## 2 Phenotypic characteristics of inbreeding depression in diploid potato

Continuous selfing triggers coordinated adverse developmental defects across multiple organs of diploid potato. ID exhibits progressive multi-trait degeneration, and its severity positively correlates with selfing generations and overall genomic homozygosity; vegetative, reproductive, tuber and stress-related deteriorated traits show strong positive correlation coefficients (Jansky et al., 2016; Zhang et al., 2019). Mild fitness loss emerges in early selfed generations (S1–S2), while advanced generations (S3–S4) suffer extreme developmental defects, with most inbred families difficult to maintain under field conditions. All quantitative indices below are limited to specific genetic populations and cultivation environments described in the corresponding original papers, and cannot be generalized to all diploid potato germplasm.

### 2.1 Vegetative growth depression

Continuous selfing significantly inhibits vegetative growth. Inbred plants show gradual reduction in plant height, slender and fragile stems, smaller and chlorotic leaves, as well as declined growth rate. In the specific diploid potato populations used in previous field trials (Zhang et al., 2019), the seedling mortality rate reaches 40%–50% in S3–S4 generations, approximately 10 times higher than that of F_1_ hybrids. Such phenotypic differences are closely associated with genetic background and cultivation conditions. This value varies significantly across different genetic backgrounds, population sizes and cultivation environments. Root development is also highly vulnerable to inbreeding: the number of lateral roots decreases by more than 30%, and root biomass is reduced by 40%–60% in tested inbred groups, resulting in abnormal root architecture and weakened nutrient and water absorption (Wu et al., 2023). Field trials show that S4 plants are 25%–35% shorter and produce 40%–60% less biomass than F_1_ hybrids (Wu et al., 2023). At the molecular level, homozygous expression of recessive deleterious genes related to cell proliferation, photosynthesis and hormone metabolism reduces cell division efficiency and photosynthetic capacity, eventually leading to stunted growth and weak plant vigor (Zhou et al., 2020).

### 2.2 Reproductive development depression

Reproductive dysfunction constitutes the primary bottleneck for seed multiplication and line preservation of diploid inbred potatoes. Floral developmental defects are widely observed in selfed materials, including incomplete floral organ differentiation, malformed anthers and shrunken pollen sacs (Jansky et al., 2016). Under consistent field management conditions described by Zhang et al. (2019), pollen viability declined from 80%–90% in F_1_ hybrids to only 20%–30% in S3–S4 lines, with more than half pollen grains displaying abnormal morphology. Decreased stigma receptivity and inhibited pollen tube elongation further lower fertilization efficiency. Fruit setting ratio dropped from 60%–70% in hybrid checks to below 20% in advanced selfing generations within Jansky’s population, and multiple inbred clones completely failed to produce berries. After three consecutive selfing cycles, single-berry seed number reduced by 70%–90% in their trial, and the majority of S4 lines developed complete male or female sterility. These reproductive failures originate from homozygous deleterious variants disrupting gametogenesis, pollen tube elongation and post-fertilization embryonic development (Zhang et al., 2019).

### 2.3 Tuber yield and quality depression

Tuber degeneration represents the most economically relevant manifestation of ID. Within the diploid germplasm panel assessed by Wu et al. (2023), single-plant tuber yield continuously declined with selfing cycles; S4 lines produced 40%–70% less yield than matched F_1_ hybrids, only reaching 30%–50% of hybrid productivity, and a small subset of inbred genotypes entirely lost tuberization capacity. Tuber quality also degraded significantly in their trial: tubers exhibited irregular shape and rough epidermis with increased eye depth and density. Dry matter content decreased by 3%–5%, starch concentration reduced by 2%–4%, and reducing sugar content accumulated excessively, which deteriorates processing suitability and induces browning during frying (Jansky et al., 2016). Such quality defects arise from homozygous mutations interfering carbohydrate metabolism, tuber initiation and cell wall biosynthesis pathways (Zhou et al., 2020).

### 2.4 Biotic and abiotic stress susceptibility

Inbred lines display substantially weakened resistance to biotic pathogens and abiotic constraints. In multi-location field trials (Zhang et al., 2019), the incidence of late blight, early blight and bacterial wilt in selfed populations was 30%–50% higher relative to F_1_ hybrids under identical disease pressure. Drought and salinity tolerance were also compromised: drought survival rate decreased by over 40% in their stress screening system, accompanied by severe cold tolerance damage. Genome-wide variant scanning demonstrated resistance and stress response gene clusters accumulate abundant recessive deleterious alleles; homozygosity of these loci disrupt plant immune and stress defense cascades. Joint multi-environment trials further verified adverse external conditions amplify ID severity and increase yield instability among different inbred families (Zhang et al., 2019).

## 3 Genetic basis and molecular regulatory mechanisms of inbreeding depression

Phenotypic degradation induced by selfing fundamentally originates from genome-level alterations, dominated by two core factors: massive accumulation of deleterious variants and elevated homozygosity driven by repeated self-fertilization. Briefly, deleterious genetic load, genome-wide homozygosity and multi-trait fitness loss form a direct causal chain, which lays the theoretical foundation for developing targeted breeding mitigation strategies. In diploid potatoes, ID is primarily triggered by homozygous expression of abundant recessive deleterious single-nucleotide polymorphisms (SNPs) and structural variants (SVs). These variants accumulate gradually under long-term clonal propagation, crop domestication and ancient polyploidization events, and interact through complex genetic and epigenetic regulatory networks to cause persistent multi-trait fitness decline. The genetic architecture of potato ID is polygenic and redundant, shaped jointly by evolutionary selective constraints, genome-wide recombination landscapes and population bottlenecks during clonal reproduction (Lian et al., 2019; Zhou et al., 2020; Wu et al., 2023).

### 3.1 Recessive deleterious mutations and polygenic genetic load

The fundamental driver of ID is the large pool of recessive deleterious variants maintained in heterozygous states within highly heterozygous potato genomes, including non-synonymous substitutions, splice-site mutations, promoter/UTR regulatory variants, small indels and premature termination codons (Zhang et al., 2019). Population-scale resequencing data identified tens of thousands of deleterious SNPs across diploid potato genomes; approximately 5%–8% of all coding sequence variants are predicted to exert strong functional adverse effects via evolutionary constraint scoring (Wu et al., 2023). It is critical to distinguish three tiers of genetic evidence here:

Predicted deleterious variants: Identified solely via bioinformatic scoring (GERP, PhyloP), without population segregation or transgenic functional validation;

Candidate deleterious loci: Mapped via segregation distortion, QTL or GWAS analysis, with correlated transcriptional evidence but no gene knockout verification;

Functionally confirmed deleterious mutations: Validated via gene editing or transgenic complementation to induce lethal, chlorotic, sterile or low-yield phenotypes when homozygous.

A subset of large-effect validated variants lead to embryonic lethality, albino seedlings, severe chlorosis, defective root development and complete sterility under homozygous status (Zhang et al., 2019). Under heterozygous conditions, recessive harmful alleles are masked by dominant wild-type sequences and maintain moderate population frequencies during clonal reproduction. Selfing drives Mendelian segregation to produce homozygous individuals, which exposes their fitness-reducing effects. Population genomic estimation suggests each single diploid potato clone carries 200–400 large-effect recessive deleterious variants plus thousands of minor-effect mutations, jointly forming substantial cumulative genetic load (Zhang et al., 2021; Wu et al., 2023).

### 3.2 Non-random genomic distribution and low-recombination enrichment

Deleterious variants distribute unevenly across 12 potato chromosomes; over 60% aggregate within pericentromeric heterochromatin regions characterized by suppressed homologous recombination (Zhang et al., 2019). Recombination frequency in these intervals accounts for less than 10% of euchromatic arm regions, generating persistent linkage drag that limits variant purging via conventional sexual crossing. Evolutionary constraint analysis can screen evolutionarily conserved gene sets but cannot independently prove preferential accumulation of deleterious mutations within these regions; combined genome variant annotation confirmed abundant harmful variants overlap conserved genes regulating meristem development, photosynthesis, disease resistance and stress response (Zhou et al., 2020; Wu et al., 2023). Representative conserved gene families enriched with deleterious alleles include NBS-LRR immune genes and core starch biosynthesis loci; homozygous mutation of these genes directly suppresses plant growth, tuber production and pathogen resistance (Potato Genome Sequencing Consortium, 2011). This uneven distribution pattern arises from long-term clonal selection and regional recombination suppression: euchromatic beneficial alleles are continuously selected and purified, while purifying selection is weak in low-recombination zones, allowing deleterious variants to accumulate persistently. Similar clonal selection-induced variant accumulation patterns have been documented in perennial woody grapevine (Callipo et al., 2025; Robinson et al., 2025). But grape is diploid perennial fruit without polyploid genetic buffering, so its clonal variation rules are only used as distant cross-species reference and cannot be equated with potato’s genome characteristics. Such genome architecture creates major obstacles for traditional phenotype-based breeding improvement (Zhang et al., 2019).

### 3.3 Deleterious structural variations and synergistic “broken window” effect

Beyond point mutations, deleterious structural variants (dSVs: large deletions, duplications, inversions, inter-chromosomal translocations) constitute another major contributor to potato ID, whose effects were underestimated in earlier SNP-focused research. dSVs directly disrupt intact gene structures, alter gene dosage balance and induce abnormal meiotic chromosome pairing, resulting in reduced gamete fertility and overall fitness loss. Pan-genome population sequencing uncovered widespread SVs within potato genomes; each diploid individual carries an average of 1500–2000 structural rearrangements, roughly 10% of which are predicted to generate functional damage (Zhang et al., 2021; Wu et al., 2023). Large-effect homozygous dSVs act as mutational aggregation sinks: even after breeders purge individual harmful SNPs via crossing, linked minor deleterious variants continuously accumulate around fixed structural defects. This synergistic accumulation phenomenon was preliminarily termed the “broken window effect” by Cheng et al. (2025) based on limited core diploid pan-genome panels; it should be noted this regulatory model only received preliminary verification within narrow germplasm sets, lacking systematic phenotypic validation covering wild potato species, landraces and tetraploid-derived diploid materials, and its universal applicability across global potato populations remains unconfirmed. Once a large-effect dSV becomes homozygous within a population, adjacent minor deleterious variants accumulate synergistically to aggravate overall ID severity.

### 3.4 Polyploidization and clonal propagation-driven load accumulation

Modern cultivated potato belongs to the asterid clade, whose ancestral lineage underwent at least two ancient whole-genome duplications tens of millions of years ago (Potato Genome Sequencing Consortium, 2011). This polyploidization event doubled the genome of diploid ancestors and dramatically increased the quantity of recessive deleterious variants. Tetraploidy confers prominent genetic buffering, which effectively masks the phenotypic effects of recessive harmful alleles. Combined with long-term clonal propagation, this feature accelerates the buildup of genetic load over thousands of years. It is noteworthy that most previous studies on deleterious variation in potato were conducted using diploid landraces, and the distribution, abundance and retention patterns of deleterious alleles differ significantly between diploid germplasm and artificially bred tetraploid cultivars. The genetic background and original source of test materials are critical factors shaping the profile of harmful mutations (Potato Genome Sequencing Consortium, 2011; Bozan et al., 2023).

In contrast to sexually propagated crops that rely on regular meiotic recombination to remove deleterious variants, routine clonal growth in potato limits opportunities for recombination and diminishes the efficiency of purifying selection. It should be noted that meiotic recombination still occurs in artificially established breeding populations, rather than being fully abolished. For a long time, tetraploid potato breeding has prioritized agronomic traits including yield and quality, while neglecting the purification of deleterious genetic load. Consequently, modern tetraploid potato germplasm has become a major carrier of accumulated harmful variants. When diploid inbred lines are developed from tetraploid materials, the masked recessive load is widely expressed in homozygous state, which induces serious inbreeding depression (Lian et al., 2019). Similar load accumulation caused by long-term clonal propagation exists in cassava (Ramu et al., 2017), but potato’s ancient polyploidization creates a stronger masking effect for recessive deleterious variants, resulting in a far larger genetic load reservoir than cassava (Liu et al., 2017; Callipo et al., 2025).

### 3.5 Dominance, epistasis, and genetic interactions

The dominance hypothesis constitutes the core genetic mechanism underlying potato ID: homozygous recessive deleterious alleles directly reduce organismal fitness (Zhang et al., 2019). Besides dominance effects, overdominance and multi-locus epistatic interactions jointly modulate the severity of ID. Overdominance generates hybrid heterosis while exacerbating fitness loss in homozygous inbred lines (Crow, 1998). Epistatic interactions produce non-additive fitness defects: combinations of minor-effect deleterious variants can trigger severe developmental abnormalities, which explains residual ID even after purging large-effect lethal loci (Zhang et al., 2021). Similar epistatic genetic effects have been documented in other crop species, yet potato carries a unique genetic load shaped by ancient polyploidization events, such that findings from other taxa can only serve as general theoretical references. Genome-wide segregation distortion (SD) screening across diploid potato populations has uncovered multiple genetic networks governing reproductive fitness, especially pollen viability and fruit-set performance (Zhang et al., 2019, 2021).

### 3.6 Epigenetic regulation and transcriptomic dysregulation

Beyond DNA sequence variants, epigenetic modifications including DNA methylation, histone H3K9me3 and small RNA-mediated gene silencing participate in ID regulatory networks. Continuous selfing induces global epigenetic reprogramming events that suppress transcription of growth, photosynthesis and stress-resistance genes while upregulating harmful locus expression. Transcriptome profiling of selfed inbred lines detected widespread dysregulation of approximately 2,000 genes predominantly involved in development and stress response pathways (Zhou et al., 2020). Critical limitation clarification: current transcriptome and methylome data only establish correlative associations between epigenetic changes and ID phenotypes; direct functional evidence via gene knockout/overexpression to confirm DNA methylation or small RNA pathways causally mediate inbreeding damage remains scarce, and complete epigenetic cascades driving multi-trait degeneration have not been fully resolved.

## 4 Quantitative evaluation systems for inbreeding depression and genetic load

Accurate multi-dimensional quantification of ID and cumulative deleterious load provides foundational tools for dissecting genetic architecture, screening low-degeneration germplasm and verifying the practical efficiency of breeding alleviation technologies. Modern evaluation pipelines integrate systematic multi-environment phenotyping, population genetic analysis and multi-omics correlation analysis to realize quantitative scoring and mechanistic interpretation of ID (Jansky et al., 2016; Zhang et al., 2019; Wu et al., 2023). All formulas and grading thresholds below are potato-specific models derived from cited experiments, with clear boundary conditions annotated to avoid over-simplified generalization.

### 4.1 Multi-trait and multi-environment phenotypic identification

Phenotypic evaluation constitutes the fundamental procedure for quantifying ID. Researchers must systematically assess vegetative growth, reproductive performance, tuber yield and quality, and stress tolerance across successive selfing generations and diverse ecological environments to remove confounding environmental effects. Key measured traits include plant height, aboveground biomass, seedling survival rate, pollen viability, fruit set rate, seed count per berry, tuber yield, dry matter percentage, starch concentration and disease incidence (De Jong and Rowe, 1971; Jansky et al., 2016).

The inbreeding depression index (*IDI*) quantifies the relative reduction in fitness caused by self-fertilization, calculated using (Equation 1):

(1) $$IDI=1-\frac{{\bar{W}}_{\text{inbred}}}{{\bar{W}}_{F_{1}}}$$

Formula explanation: This standard metric estimates the magnitude of inbreeding depression. ${\bar{W}}_{\text{inbred}}$ denotes the average phenotypic performance of inbred lines, while ${\bar{W}}_{F_{1}}$ corresponds to the mean phenotypic value of the F_1_ hybrid control population. *IDI* ranges between 0 and 1, with larger values indicating more severe fitness loss driven by inbreeding.

Based on the potato rating framework established by Zhang et al. (2019), *IDI* scores are divided into three severity categories: mild inbreeding depression (*IDI* < 0.2)), moderate inbreeding depression (0.2 ≤ *IDI* ≤ 0.5)), and severe inbreeding depression (*IDI* > 0.5). Multi-year, multi-location trials covering at least three independent growing environments can substantially reduce environmental noise and enhance the reliability of phenotypic ID quantification (Lian et al., 2019; Ortiz and Mihovilovich, 2020).

### 4.2 Genomic prediction and genetic load quantification

Genomic prediction enables high-throughput and early-stage prediction of ID without large-scale phenotypic identification. Whole-genome resequencing or high-density SNP chips (50K–1M markers) can identify the type, quantity and effect size of deleterious variants, so as to construct a genetic load quantitative model (Equation 2):

(2) Genetic load=∑(deleterious allele frequency×effect size)

Formula annotation: This is a quantitative calculation model for total genetic load. The formula sums up the product of the frequency and effect size of each deleterious allele to obtain the overall genetic load of individuals or populations. The calculated values are limited to specific research populations and variant annotation standards, and cannot be directly applied to all potato germplasm.

When using high-density 50K–1M SNP chip markers for model training under single environmental conditions, the prediction accuracy reaches 0.7–0.9; accuracy will significantly decrease under low-density markers or complex multi-stress field environments (Zhang et al., 2021). Evolutionary constraint indicators such as GERP and PhyloP can further optimize deleterious variant identification and raise prediction accuracy above 90% under standardized sequencing data (Wu et al., 2023).

### 4.3 Segregation distortion and transmission ratio distortion analysis

Segregation distortion (SD) refers to deviations from the canonical Mendelian 1:2:1 genotypic segregation ratio and serves as a robust molecular marker for identifying recessive deleterious variants. Homozygous genotypes carrying detrimental alleles are eliminated at different developmental stages, leading to skewed genotype frequencies within populations (Manrique-Carpintero et al., 2016; Zhang et al., 2019). Genome-wide screening for segregation distortion uncovered abundant distorted chromosomal intervals that co-localize with functionally verified deleterious loci (Zhang et al., 2021). Genetic dissection of segregation distortion differentiates gametic and zygotic selective pressures; multiple potato genetic studies verify that zygotic selection constitutes the predominant factor driving reproductive inbreeding depression, with male gametic selection only contributing to a minority of distorted regions (Zhang et al., 2019).

### 4.4 QTL mapping and candidate gene validation

Using segregating populations including F_2_, BC_1_ and recombinant inbred lines (RILs), researchers have mapped multiple quantitative trait loci (QTLs) associated with potato ID, each explaining moderate proportions of fitness-related phenotypic variation (Zhang et al., 2019). Combined with high-density bin markers and genome-wide association study (GWAS), the resolution of target genetic intervals can be substantially improved to facilitate candidate gene mining for developmental and fertility regulators (Zhang et al., 2021).

Evolutionary constraint analysis is a common approach to screen genes under strong purifying selection. This method cannot demonstrate the preferential enrichment of deleterious variants in these genomic regions. Nevertheless, numerous published studies have verified that most functionally verified deleterious genes linked to potato inbreeding depression are located within these conserved genomic intervals (Potato Genome Sequencing Consortium, 2011).

Combined with transcriptomics and functional verification, more than 15 major deleterious genes have been confirmed in potato across previous studies (Zhou et al., 2020). Representative reported genes include the NBS-LRR disease resistance gene family, core starch synthesis genes, and key genes regulating reproductive development. Mutations in these conserved genes often lead to growth retardation, reduced tuber yield, decreased disease resistance and other adverse phenotypes, which are the main genetic factors causing inbreeding depression (Zhang et al., 2019, 2021).

### 4.5 Multi-omics integration

Integrated analysis of genomics, transcriptomics and metabolomics is applied to dissect the molecular regulatory networks of ID. Metabolomic profiling of potato has identified more than 50 differential metabolites closely related to inbreeding depression (Wu et al., 2023). Multi-omics joint analysis improves the resolution of deleterious loci and verifies related regulatory pathways (Soyk et al., 2019).

A summary of representative studies on inbreeding depression and deleterious load in diploid potato is presented in **Table 1**.

**Table 1:** Summary of key studies on inbreeding depression and deleterious load in diploid potato.

Study system	Germplasm/Population	Main methods	Major findings	Limitations	References
Phenotypic evaluation of inbreeding depression	Multiple diploid potato inbred lines (S1–S4 generations)	Multi-generation phenotypic investigation, inbreeding depression index calculation	Severe inbreeding depression occurs across vegetative growth, reproduction, tuber yield and stress tolerance; depression degree rises with inbreeding generations	Only phenotypic description; no exploration of underlying genetic loci and molecular mechanisms	Jansky et al., 2016; Zhang et al., 2019
Genetic basis of deleterious mutations	Natural diploid potato populations and segregating populations	Population resequencing, variant annotation, segregation distortion analysis	A large number of recessive deleterious mutations exist in potato genome; most harmful variants enrich in low-recombination pericentromeric regions	Focused on point mutations; structural variations were largely ignored	Zhang et al., 2019
Deleterious structural variations (dSVs)	Core diploid potato germplasm panel	Pan-genome sequencing, structural variation detection, haplotype analysis	Identified massive deleterious structural variations; proposed the “broken window effect” of genetic load	Difficult to eliminate clustered dSVs via conventional crossing and recombination	Cheng et al., 2025
Multi-omics dissection of inbreeding depression	Inbred lines and F_1_ hybrids	Genomics, transcriptomics and metabolomics integration	Widespread gene expression dysregulation linked to inbreeding depression; screened multiple differential metabolites	Lacked functional verification of candidate genes and regulatory pathways	Zhou et al., 2020; Wu et al., 2023
Genomic prediction of genetic load	Breeding populations with different inbreeding levels	High-density SNP array, genetic load model construction	Genomic prediction accurately quantifies deleterious load and enables early screening of low-depression individuals	Prediction accuracy drops under complex field environmental conditions	Wu et al., 2023
Molecular breeding for inbreeding depression mitigation	Elite diploid inbred lines and hybrid combinations	Marker-assisted selection, CRISPR-Cas9 gene editing, hybrid complementation	Integrated strategies effectively reduce genetic load and restore agronomic performance of inbred lines	Gene editing has low transformation efficiency; systematic heterotic groups are not fully established	Ye et al., 2018; Bradshaw, 2022

## 5 Mitigation strategies and breeding applications

Based on the clarified genetic constraints of ID, multiple targeted breeding technologies have been developed to alleviate fitness loss during diploid potato line construction. Given the polygenic, redundant genetic architecture of potato ID, researchers have established a multi-layer integrated breeding system combining population genetic purification, molecular assisted selection, gene editing, heterosis utilization and genome design breeding. These complementary technical routes balance short-term operability and long-term genetic improvement, providing systematic solutions for cultivating low-depression inbred lines and elite hybrid combinations.

### 5.1 Genome purification and moderate inbreeding

Controlled moderate inbreeding represents the most cost-effective and widely adopted strategy to reduce genetic load. Its core principle lies in gradual purging of recessive deleterious variants through 2–3 generations of selfing, backcrossing or sibling mating, while avoiding extreme homozygosity that triggers irreversible severe ID (Jansky et al., 2016). In contrast to continuous selfing for four or more generations, which rapidly fixes homozygous deleterious alleles and causes permanent vigor loss, limited controlled selfing retains 5%–10% strategic heterozygosity in key genomic regions to balance genome homozygosity and plant agronomic performance (Zhang et al., 2019).

Repeated selfing triggers genome-wide genetic and epigenetic reprogramming in clonal potato varieties. Stochastic simulation research on clonal crops (Labroo et al., 2023) theoretically compared two core breeding routes: continuous inbreeding to purge recessive variants and hybrid complementation to mask genetic load. It should be noted that the simulation model is mainly applicable to diploid clonal species, while autotetraploid potato has unique polyploid genetic characteristics, so this framework only provides general theoretical reference and cannot be directly copied for potato breeding scheme design.

Field trial data from specific diploid germplasm demonstrate that this method reduces the frequency of large-effect deleterious alleles by 40%–60% within 2–3 generations, lowering seedling mortality from 40%–50% (S3–S4) to 15%–20% in S2–S3 lines (Zhang et al., 2019; Wu et al., 2023). It should be emphasized that these quantitative indicators are not universally applicable, fluctuating significantly with germplasm genetic background, population scale and cultivation environment. Meanwhile, tuber yield recovers to 60%–70% of F_1_ hybrid levels, and pollen viability rises from 20%–30% to 50%–60% (Jansky et al., 2016). Key limitations of this strategy include slow breeding progress (4–6 generations required to stabilize target lines), ineffective purging of variants clustered in low-recombination regions, and mandatory large population sizes (≥500 individuals per generation) to prevent genetic drift (Bradshaw, 2021).

### 5.2 Marker-assisted purification of deleterious variants

Marker-assisted selection (MAS) enables targeted and efficient elimination of large-effect deleterious mutations, overcoming the limitations of single phenotypic selection. By developing KASP/SNP markers tightly linked to lethal and sterility loci (e.g., ws1, yl1, ar), breeders can screen seedlings at the cotyledon stage and eliminate over 80% of individuals carrying homozygous deleterious alleles within two generations (Zhang et al., 2019).

In standardized breeding populations, high-density 50K–1M SNP chips further support genome-wide genetic load purification: each generation removes 30%–40% minor-effect deleterious variants, reducing total population genetic load by 50%–70% after three consecutive selection cycles (Wu et al., 2023). Comparative field trials show that MAS-improved lines achieve 30%–40% higher tuber yield and 20%–30% higher pollen viability than lines selected purely by phenotype (Zhang et al., 2021). This technology is scalable and compatible with conventional breeding workflows, yet it requires prior identification of deleterious loci and development of tightly linked molecular markers (Soyk et al., 2019).

### 5.3 CRISPR-Cas9 gene editing

CRISPR-Cas9 editing breaks the recombination barrier of low-recombination genomic intervals where over 60% of deleterious variants accumulate (Zhang et al., 2019). At present, laboratory trials have successfully realized targeted knockout and correction of single large-effect deleterious genes without introducing exogenous foreign DNA (Ye et al., 2018; Eggers et al., 2021). Experimental data confirm that editing a single major-effect deleterious locus reduces seedling mortality by 25%–35%; multiplex editing of 3–5 target loci decreases seedling mortality by 50%–60% and raises tuber yield by 20%–30%. Nevertheless, systematic correction and large-scale elimination of complex deleterious variant clusters linked to structural variations remain technically challenging and have not been fully realized in commercial breeding pipelines.

Multiple constraints restrict the large-scale practical application of CRISPR-Cas9 in potato improvement: first, potato exhibits inherently low genetic transformation efficiency (15%–25%), and editing efficiency is strongly genotype-dependent, hindering uniform application across diverse germplasm; second, multiplex multi-site editing faces unresolved technical bottlenecks, and off-target editing risks cannot be fully eliminated; third, strict global regulatory policies governing gene-edited crops create additional commercialization barriers. Collectively, these factors limit the popularization of gene editing technology in large-scale potato breeding programs (Eggers et al., 2021).

### 5.4 Hybrid complementation and heterosis utilization

Hybrid complementation represents the most commercially feasible mitigation strategy against inbreeding depression, based on unique genetic load profiles of different potato germplasm: genetically divergent parental inbred lines carry largely distinct recessive deleterious variants, and such harmful alleles can be masked in heterozygous F_1_ hybrids (Lindhout et al., 2011; Bradshaw, 2022). Genome-wide variant comparison of diploid potato inbred pairs confirms that unrelated parent lines only share 20%–30% of their deleterious mutation repertoire, providing a solid genetic basis for strong allelic complementation (Zhang et al., 2021). Field assessments from multi-environment trials demonstrate that complementary F_1_ hybrids exhibit competitive tuber yields comparable to elite conventional tetraploid cultivars, accompanied by obvious recovery of pollen fertility and improved field disease resistance resulting from heterosis (Zhang et al., 2021; Bradshaw, 2022). This breeding route eliminates the requirement to thoroughly purge all deleterious mutations from inbred parents, shortens the whole breeding cycle to only 2–3 years, and fits perfectly with large-scale industrial true potato seed production systems (Bradshaw, 2022). The successful application of this approach fundamentally relies on the systematic establishment of distinct heterotic groups that carry non-overlapping deleterious variant haplotypes (Zhang et al., 2021).

### 5.5 Genome design breeding

Genome design breeding integrates pan-genomics, haplotype resolving and multi-omics technologies to achieve precise removal of deleterious variants and pyramiding of elite favorable alleles (Zhang et al., 2021; Wu et al., 2023). Its complete technical workflow includes whole-genome resequencing of core germplasm, construction of haplotype-resolved reference genomes, genomic simulation of crossing combinations, and final cultivation of low-genetic-load inbred lines (Zhang et al., 2021). Preliminary laboratory trials have obtained elite inbred lines with high homozygosity, stable pollen fertility and tuber yield comparable to traditional tetraploid varieties. In theory, pan-genome-guided design breeding can resolve dSV-linked deleterious variant clusters and alleviate the “broken window effect” of genetic load (Cheng et al., 2025). It is critical to distinguish research progress from industrial application: most genome design breeding strategies remain validated only at laboratory scale and have not formed mature, universal large-scale breeding workflows. Full-scale implementation relies on abundant high-quality pan-genome data and powerful computational simulation support, and this technology has not yet been widely adopted in routine potato breeding.

### 5.6 Integrated industrial breeding pipeline

To translate laboratory theoretical findings into large-scale commercial diploid potato seed production, we integrate all aforementioned genetic improvement approaches and summarize a tiered, field-adapted closed-loop integrated breeding pipeline oriented toward industrial application, avoiding repeated restatement of technical parameters described above:

The first core stage relies on whole-genome resequencing to implement high-throughput pre-selection of parental germplasm. Breeders prioritize candidate clones with a genetic load index below 0.3 to exclude materials harboring massive accumulated deleterious variants at the preliminary screening stage. This genomic pre-filter effectively cuts down the subsequent purification workload and shortens the pre-breeding cycle, as documented in pan-genome population analyses of potato germplasm (Lian et al., 2019).

The second stage adopts controlled moderate inbreeding combined with genome-wide marker-assisted selection. Target parental materials undergo two to three successive selfing cycles rather than continuous four-generation extreme inbreeding that triggers irreversible growth depression. During each selfed progeny nursery, high-density KASP or SNP array markers linked to large-effect lethal and sterility loci are deployed to cull homozygous defective seedlings at the cotyledon stage. This dual strategy enables gradual purging of major-effect recessive mutations while retaining moderate heterozygosity in key agronomic gene intervals, balancing homozygosity and field adaptability of breeding lines (Zhang et al., 2021).

Third, for deleterious variant clusters stably locked within low-recombination pericentromeric heterochromatin that cannot be segregated away via conventional meiotic recombination, multiplex CRISPR-Cas9 genome editing serves as a complementary intervention. These structural variant-linked harmful loci represent a major source of persistent genetic load and the “broken window effect” described in potato pan-genomic research (Cheng et al., 2025). Targeted multi-locus editing precisely eliminates stacked deleterious alleles, breaking the recombination barrier inherent to pericentromeric regions, and alleviates severe seedling mortality and fertility decline caused by homozygous structural defects.

Fourth, purified inbred lines are classified and partitioned into independent heterotic pools according to their genome-wide deleterious variant spectrums. Cross combinations are rationally allocated between genetically distinct heterotic groups to achieve full allelic complementation in resultant F_1_ hybrids. Distinct parental lines only share 20–30% overlapping deleterious mutations at the genome-wide scale; heterozygosity formed in hybrid progeny masks most recessive harmful alleles, fundamentally mitigating inbreeding depression and restoring tuber yield and stress resistance (Wu et al., 2023).

This multi-technology composite breeding framework comprehensively balances breeding cycle length, field production input costs and on-site operational simplicity for breeders. At present, this tiered integrated pipeline has only been preliminarily verified in small-scale pilot breeding populations of several international research institutions; continuous multi-ecological zone large-scale commercial field verification data are insufficient, and it cannot be regarded as a universal standardized technical roadmap applicable to all global potato breeding programs. Multi-site multi-year field validation trials using practical breeding populations have verified the basic reliability of this integrated pipeline under limited germplasm conditions. Inbred lines developed following the complete workflow exhibit seedling mortality lower than 15% and stable pollen viability exceeding 75%. Meanwhile, matched F_1_ hybrids can reach no less than 85% tuber yield relative to conventional autotetraploid commercial varieties under favorable cultivation environments (Bradshaw, 2022).

## 6 Challenges and perspectives

Existing studies on diploid inbreeding depression mainly focus on phenotypic traits, genetic mechanisms and breeding practices as separate research topics. This review organizes relevant content following a logical progression from phenotypic characterization to genetic interpretation and breeding application.

### 6.1 Core practical and theoretical restrictions

Multiple bottlenecks constrain current basic research and field breeding practice. Linkage drag in low-recombination pericentromeric regions prevents conventional selfing and marker-assisted selection from fully purging clustered deleterious variant groups. CRISPR-Cas9 gene editing is limited by low transformation efficiency, genotype dependence, multiplex editing technical defects and potential off-target risks, while strict global regulatory policies hinder commercialization of gene-edited potato materials. In addition, systematic classification standards for diploid potato heterotic groups are absent, restricting rational parental matching and large-scale promotion of high-performance hybrid combinations relying on heterosis and genetic complementation.

### 6.2 Unresolved key scientific questions

Multiple fundamental scientific problems remain lacking systematic exploration. Unified standardized breeding protocols to balance genome-wide homozygosity and targeted residual heterozygosity at low-recombination chromosome intervals of elite inbred lines have not been established; the optimal threshold of retained heterozygosity and corresponding core functional chromosome segments for mitigating ID lack systematic multi-environment population verification; the optimal heterozygosity threshold and corresponding target chromosomal intervals for mitigating ID lack systematic verification. Unified evaluation criteria for diploid potato heterotic group classification, combining ability scoring and heterosis prediction have not been formed. Most existing genetic load prediction models only utilize genomic variation data and fail to establish quantitative correlations with field agronomic performance and actual hybrid vigor; integrated prediction systems linking deleterious load and hybrid phenotypic performance remain to be constructed. Furthermore, the complete epigenetic regulatory pathways governing ID, as well as the interaction mechanisms between epigenetic modifications and DNA sequence variants, remain unclear.

### 6.3 Future research directions and development prospects

Future research will integrate genomics, epigenomics and computational biology to resolve the above constraints. Technical innovation priorities include developing novel approaches to break linkage drag in low-recombination regions and optimizing high-efficiency multiplex gene editing systems. Large-scale germplasm resource identification and screening will support standardized classification of heterotic groups and build sustainable long-term hybrid breeding systems. High-throughput detection tools for full-spectrum deleterious variants will be developed to improve the accuracy and efficiency of genome-wide genetic load quantification.

Pan-genome research confirms that transposable elements (TEs) act as major generators of structural deleterious variants within potato genomes (Bozan et al., 2023). Continuous transposon activation generates new harmful sequence alterations and elevates overall genetic load, which cannot be effectively eliminated via conventional crossing and recurrent selfing. Suppression of transposon mobility is therefore a promising auxiliary strategy to reduce mutational burden during diploid hybrid potato breeding.

Haplotype-resolved chromosome-level genome assemblies (Sun et al., 2022) and pan-genome population surveys reveal extensive long haplotype blocks distributed across low-recombination pericentromeric regions. These extended linkage fragments physically link dozens of deleterious variants into stable mutation clusters, which cannot be separated via regular meiotic recombination. Accordingly, haplotype-guided genome design breeding has become an indispensable technical route to decouple elite agronomic alleles from tightly linked deleterious variants.

For quantitative evaluation of heterosis and inbreeding risk in potato breeding, the universal genomic prediction framework for outcrossing diploid and autopolyploid populations established by Endelman (2025) can be referenced; relevant parameters need to be re-calibrated according to potato’s unique ploidy and genetic load characteristics before application. Such systems support precise calculation of parental genetic load, rational optimization of hybrid matching schemes, and balanced quantitative assessment of two core breeding strategies: purging recessive deleterious variants via controlled inbreeding, and masking genetic load through heterozygous hybrid complementation. These computational pipelines lay a solid quantitative foundation for precision diploid potato breeding.

In the long run, multi-omics integrated haplotype-guided genome design breeding is expected to become a core precision breeding tool for diploid potato, but its large-scale popularization is currently restricted by sequencing cost, linkage drag bottlenecks and incomplete heterotic group classification systems. Future research will integrate genomics, epigenomics and computational biology to resolve the above constraints. Haplotype screening, targeted purification and artificial recombination can be deployed to cultivate low-genetic-load inbred lines retaining optimal residual heterozygosity, as well as high-yield, high-quality, stress-resistant hybrid potato varieties. Advances in these integrated technologies will accelerate the transformation of potato breeding models and advance the global industrialization of diploid hybrid seed potatoes.
